# A Rare Presentation of Wegener’s Granulomatosis With Perinuclear Antineutrophil Cytoplasmic Antibody Positivity

**DOI:** 10.7759/cureus.53078

**Published:** 2024-01-27

**Authors:** Dhammika P Rathnayake, KVC Janaka, Hassan Hussain, Sudeshan Senanayake, WDS Lakindi

**Affiliations:** 1 General Internal Medicine, Sri Jayawardenapura General Hospital, Colombo, LKA; 2 Internal Medicine, Sri Jayawardenapura General Hospital, Colombo, LKA; 3 Internal Medicine, University of Peradeniya, Kandy, LKA; 4 Internal Medicine, District General Hospital Nuwara Eliya, Nuwara Eliya, LKA

**Keywords:** autoimmune vasculitis, vasculitis, p-anca, granulomatosis with polyangiitis (gpa), wegeners granulomatosis

## Abstract

Wegener’s granulomatosis (WG), also termed granulomatosis with polyangiitis, is a vasculitic condition with numerous systemic manifestations. It is mainly a pauci-immune vasculitis which involves small and medium-sized vessels. Herein, we report a case of a 72-year-old female with a background history of type 2 diabetes mellitus and hypertension. She presented to the hospital with recurrent nasal congestion, headache, and intermittent facial pain for two months' duration. At the initial presentation, she was diagnosed with pansinusitis, for which she was treated with antibiotics and was discharged. Two weeks later, she presented with progressively worsening bilateral lower limb numbness and weakness for one week's duration. With serial investigations, she was diagnosed with WG and was treated with intravenous methylprednisolone along with cyclophosphamide, after which she made a significant improvement in her clinical status.

## Introduction

Wegener’s granulomatosis (WG) is a granulomatous inflammation with multisystem involvement. It can be necrotizing in nature and commonly involves the upper and lower respiratory tracts and the kidneys. In WG, other organ systems can also become involved; however, WG has a "limited form", where only a single organ involvement is seen in addition to the lungs and kidneys [1]. Neurological involvement in WG occurs in 20-50% of patients [1]. The pathogenesis of WG is not well understood, but the presence of a newly identified auto-antibody, termed antineutrophil cytoplasmic antibody (ANCA), in the majority of patients with WG suggests the possibility of an autoimmune mechanism mediating the disease process. Out of the patients with WG, the majority presents with (c)-ANCA positivity, and the presence of perinuclear (p)-ANCA positivity is sparse [1]. The main objective of this case report is to enlighten the variety of clinical presentations to come to a diagnosis even when autoantibody tests are not conclusive.

## Case presentation

**Figure 1:**
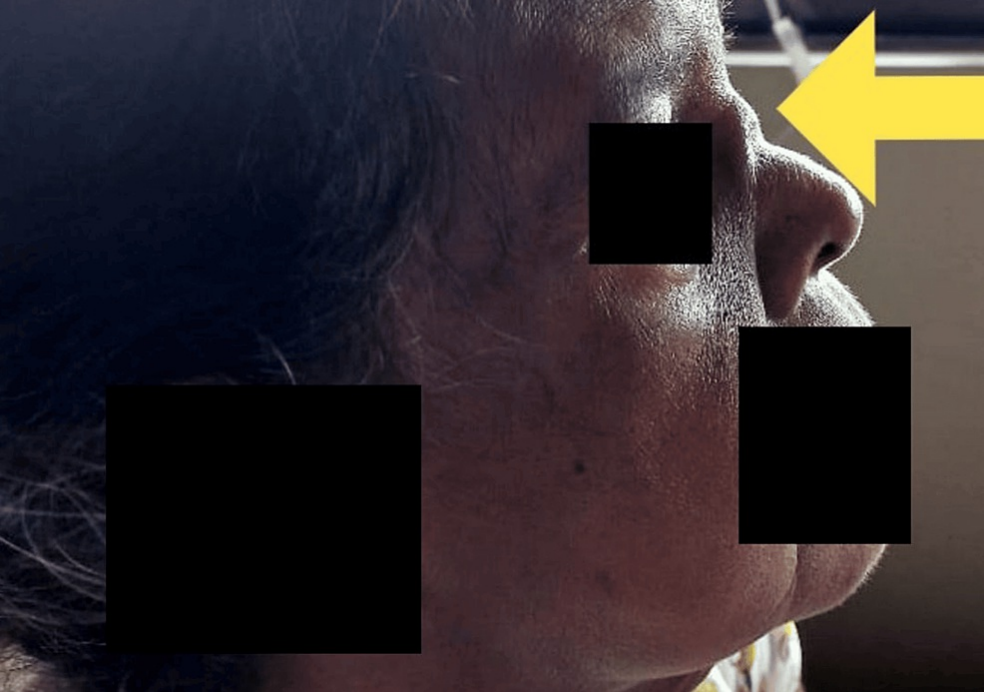
Nasal deformity: lateral view.

**Figure 2:**
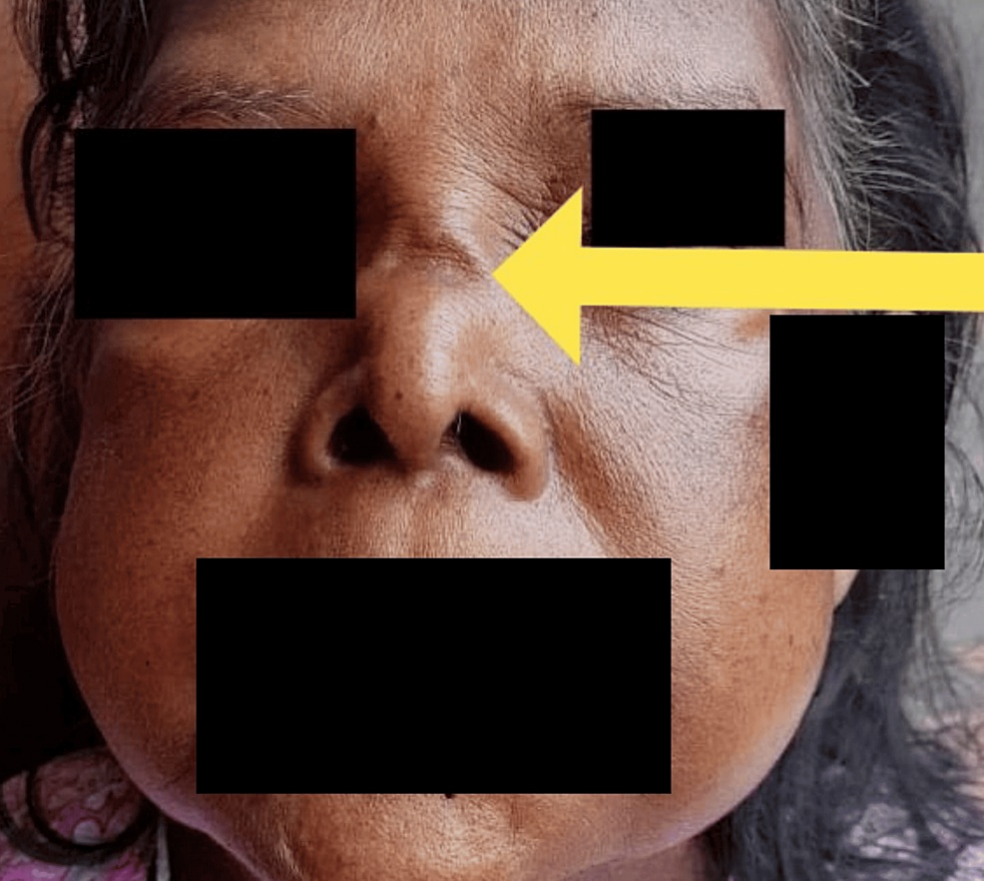
Nasal deformity: frontal view.

A 72-year-old female with a background history of well-controlled type 2 diabetes mellitus and hypertension presented to the hospital with recurrent nasal congestion, intermittent diffused headache of moderate severity, and intermittent bilateral facial pain for a two-month duration along with a history of recurrent upper respiratory tract infections. She did not have fever, cough, shortness of breath, or hemoptysis at the time of presentation. There was no history suggestive of urinary symptoms such as hematuria or froth in urine. She had noticed a recent onset nasal deformity (lateral and frontal views are shown in Figures 1, 2) but denied any neurological or skin manifestations. The rest of the systemic inquiry was unremarkable.

Upon examination, she was an averagely built lady, whose appearance was consistent with her chronological age. She was well-looking and was not in pain at the time of examination. There was an obvious deformity noted at the middle of the nasal bone, but no signs of inflammation were evident at that site. There were no other obvious deformities or facial asymmetry noted. She was afebrile, there was no conjunctival pallor or plethora. No nasal polyps were noted. There was no generalized lymphadenopathy. Her pulse rate was 82 beats per minute, regular, and normal in volume and character. Her blood pressure was 130/80 mmHg. First and second heart sounds were heard, and there were no murmurs. Her lungs were clear with normal vesicular breathing and no added sounds were present. There was no renal angle tenderness and no ankle edema present. Neurological examination was insignificant with intact motor, sensory, and coordination. The rest of the systemic examination was unremarkable.

During the current admission, basic investigations were carried out along with investigations for the said nasal cartilage deformity, but no evidence of a tumor or malignancy was found. A nasal biopsy was performed, and a slit skin smear was done to exclude leprosy. However, both studies were found to be normal. The basic blood investigations carried out revealed high inflammatory markers; hence, she was transferred to the medical unit for further evaluation. There were a few other investigations that were carried out in view of arriving at a diagnosis (Table 1). Following the investigations, she was treated for pansinusitis with intravenous and oral antibiotics and was discharged upon her request.

**Table 1:** Investigations. CRP: C-reactive protein.

Investigation		Initial Value	2 Weeks After	Reference Range
Full blood count	White cell count (× 10^3^/µL)	15.3	10.66	4–11
	Neutrophils (× 10^3^/µL)	12.09	7.79	1.5–7.5
	Hemoglobin (g/dL)	10.7	7.9	12–14
	Mean corpuscular volume (fL)	–	88	80–100
	Mean corpuscular hemoglobin (pg)	–	28.85	27–32
	Platelets (× 10^3^/µL)	386	184	150–400
Inflammatory markers	CRP (mg/L)	177	5	<5
	Erythrocyte sedimentation rate (ESR) (mm in the first hour)	–	110	<20
Renal profile	Serum creatinine (µmol/L)	94	366	53–97.2
	Serum sodium (mmol/L)	129	–	135–145
Urine full report	Pus cells (per high-power field)	3–5	2–4	
	Red cells (per high-power field)	10–12	Moderately field full	
	Red cell casts	Negative	–	
Imaging studies	Ultrasound scan of the abdomen	Grade 1 fatty liver	Normal renal morphology	

In less than two weeks, she presented again to the medical unit with progressively worsening bilateral lower limb numbness and weakness for a one-week duration. She complained of difficulty in walking but denied having fever, headache, blurring of vision, or slurred speech. During the period of her hospital stay, she developed sudden onset shortness of breath without chest pain or cough. Examination revealed bi-basal fine crepitations. She had neither vasculitic rash nor hypopigmented skin lesions. She did not have hepatosplenomegaly, and her neurological examination was unremarkable except for the newly developed lower motor neuron type right-sided foot drop.

Investigations were carried out to further evaluate her condition, and the results were as follows (Table 1). Her liver function tests were normal. Her serum C-ANCA was negative, and P-ANCA was positive. Tests for HIV 1 and 2 and hepatitis B and C infections were all negative. Her blood picture showed a reactive picture with an infective or inflammatory process, mild anemia suggestive of anemia of chronic disease, and marked rouleaux formation without eosinophilia.

**Figure 3:**
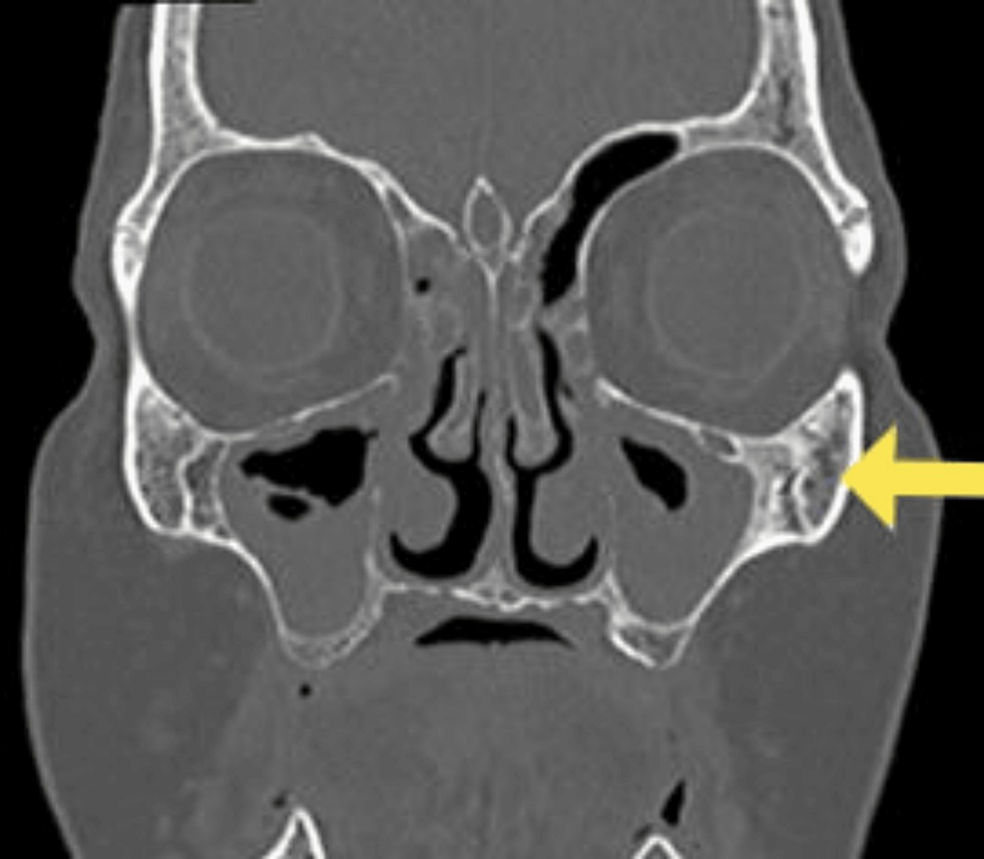
Computed tomography scan showing pan sinusitis.

**Figure 4:**
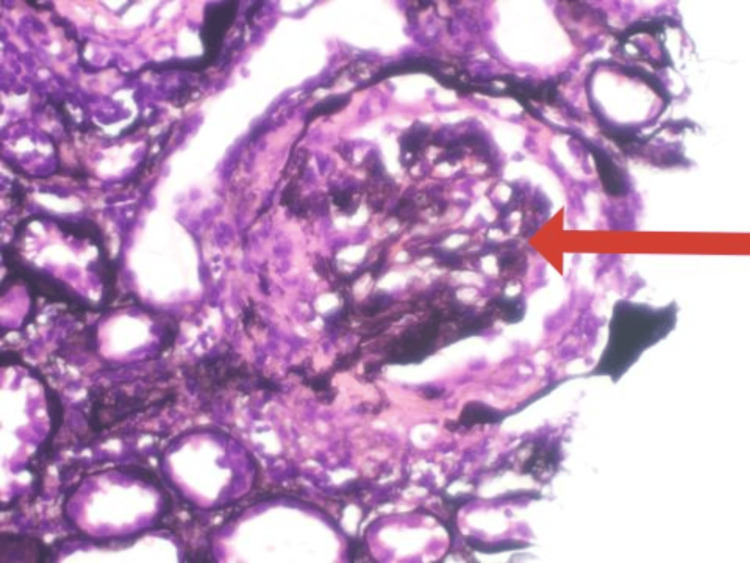
Renal biopsy showing 5/5 glomeruli with diffuse proliferative changes, cellular and fibrocellular crescents in 3/5 glomeruli, interstitial fibrosis and tubular atrophy (IFTA) of 10%, and appearances consistent with crescentic pauci-immune glomerulonephritis.

A computed tomography scan of her sinuses showed pansinusitis (Figure 3). No granulomatous changes were identified in her nasal biopsy. Renal biopsy revealed 5/5 glomeruli with diffuse proliferative changes, cellular and fibrocellular crescents in 3/5 glomeruli, interstitial fibrosis and tubular atrophy (IFTA) of 10%, and appearances consistent with crescentic pauci-immune glomerulonephritis (Figure 4). The findings of her nerve conduction study were consistent with asymmetrical sensory and motor axonal peripheral neuropathy or axonal type mononeuritis multiplex. Sural nerve biopsy was unremarkable. Slit skin smears taken from both her earlobes showed no acid-fast bacilli (AFB) to suggest leprosy.

Based on the investigations, a diagnosis of WG was made, and the patient was managed with a multidisciplinary approach involving nephrology, pathology, and neurology. She was initially started on intravenous methylprednisolone 1 g daily for three days, followed by oral prednisolone 60 mg daily. Due to the unavailability of Rituximab, she was given oral cyclophosphamide 2 mg/kg daily. She was discharged at the remission induction stage with stable renal functions and improving constitutional symptoms.

## Discussion

Granulomatosis with polyangiitis (GPA), formerly known as Wegener's granulomatosis (WG), was first described in 1933 and is one of the ANCA-associated small vessel vasculitides. Histologically, it is characterized by necrosis, granulomatous inflammation, and vasculitis [2].

GPA has a strong association with C-ANCA, which are autoantibodies directed against proteinase 3, a constituent of azurophilic granules of neutrophils [2]. C-ANCA is present in 80-90% of cases and drives the tissue injury in GPA [3,4]. However, its presence is not essential to make the diagnosis [5]. The antibody titer of C-ANCA falls dramatically with effective treatments and therefore is a useful marker of disease activity [6]. Most patients with GPA are white and middle-aged, although some patients present at extremes of age. GPA has an equal sex distribution [2]. GPA mostly affects the upper and lower respiratory tracts and kidneys, therefore has a diverse presentation leading to a number of differential diagnoses [7].

Clinical presentation includes unexplained constitutional symptoms like fever and weight loss [2]. Upper airway diseases are the most common form of presentation, including sinusitis, hearing loss, epistaxis, and saddle nose deformity [8]. Sinusitis is the most frequent initial presentation in 50% to 66% of patients with GPA. Lower respiratory tract involvement occurs in 45% of patients as an initial presentation and in 80% during the course of the illness. In addition to sinusitis, the most common pulmonary symptoms include cough, hemoptysis, and pleuritis [2]. Radiologically, pulmonary infiltrates and nodules are commonly noted [8]. Renal disease can also present as the initial presentation or during the course of the illness. Histologically, it varies from focal segmental glomerulonephritis to fulminant crescent glomerulonephritis. Even with appropriate therapy, it can result in chronic renal failure [2].

Ocular manifestations are known to occur in 29% to 79% of patients with GPA [9]. Any compartment of the eye could be affected and can result in keratitis, conjunctivitis, scleritis, episcleritis, uveitis, retroorbital pseudotumor with proptosis, retinal vein occlusion, and optic neuritis. This warrants a complete ophthalmological evaluation in patients presenting with GPA [10]. Gastrointestinal system involvement is rare and is reported only in 10% to 20% of patients. This includes upper gastrointestinal bleeding, colitis, and perforation [11]. Cardiac involvement occurs in 6% to 44% of cases. Pericarditis, myocarditis, and aortitis that may lead to malignant arrhythmias have been reported. Cardiac involvement is associated with increased mortality and morbidity in GPA [12]. Skin manifestations can present as purpura, vasculitic ulcers, or gangrenes [13]. Nervous system involvement may lead to seizures, cerebrovascular accidents, multiple cranial nerve palsies, and mononeuritis multiplex [14].

The 2022 American College of Rheumatology/European League Against Rheumatism (ACR/EULAR) classification criteria can be used to classify a patient as having GPA, and a score of ≥5 from the following ten items is required for classification (Table 2) [15]. Our patient had recurrent sinusitis and saddle nose deformity. During the course of the illness, she developed mononeuritis multiplex and pulmonary edema due to deteriorating renal functions. We also had limitations due to financial constraints in diagnostic testing and management of this patient.

**Table 2:** 2022 ACR/EULAR criteria for GPA. ACR/EULAR: American College of Rheumatology/European League Against Rheumatism, GPA: granulomatosis with polyangiitis, ANCA: antineutrophil cytoplasmic antibody.

1. Nasal involvement such as bloody discharge, ulcers, crusting, congestion, blockage and septal defect or perforation	+3
2. Cartilaginous involvement, including inflammation of ear or nasal cartilage, hoarse voice, stridor, endobronchial involvement, or saddle nose deformity	+2
3. Hearing loss: conductive or sensorineural	+1
4. Positive test for cANCA or antiproteinase 3 (anti-PR3) antibodies	+5
5. Chest imaging: pulmonary nodules, mass, or cavitation	+2
6. Biopsy: granuloma, extravascular granulomatous inflammation, or giant cells	+2
7. Imaging: inflammation, consolidation, or effusion of nasal/paranasal sinuses or mastoiditis	+1
8. Biopsy: pauci-immune glomerulonephritis	+1
9. Positive test for pANCA or antimyeloperoxidase (anti-MPO) antibodies	–1
10. Blood eosinophils ≥1×10^9^/L	–4

**Table 3:** Treatment of GPA. GPA: granulomatosis with polyangiitis, MMF: mycophenolate mofetil.

Active Severe GPA	Active Nonsevere GPA
Remission induction: rituximab over cyclophosphamide glucocorticoids	Remission induction: methotrexate over other medications such as glucocorticoids
Remission maintenance (in order of preference): rituximab methotrexate/azathioprine MMF leflunomide	Remission maintenance: continue the same medication

Her GPA classification score was six. The ACR guidelines categorize GPA into five disease states: active disease, severe disease, non-severe disease, remission, refractory disease, and relapse. In our case, the patient was experiencing active severe disease. Active disease is defined as new, persistent, or worsening signs or symptoms attributed to GPA, MPA, or EGPA and not related to prior damage. Severe disease is characterized by vasculitis with life- or organ-threatening manifestations [16].

Patients should be managed in specialist centers with a multidisciplinary approach [17]. If left untreated, GPA has an extremely poor survival rate, and the mean survival of untreated patients is five months [18]. This justifies the aggressive use of immunotherapy in the treatment of GPA. Medical management is aimed at achieving two stages: remission induction and remission maintenance. Recommended therapies depend on the disease state (Table 3) [16].

Early achievement of remission induction is important to prevent renal damage [18]. All patients with acute GPA should be treated with corticosteroids. However, monotherapy with corticosteroids is not recommended [19].

In a patient with active severe disease, remission induction is achieved by combining steroids with rituximab or cyclophosphamide. Studies show that both rituximab and cyclophosphamide have similar benefits in terms of efficacy. However, rituximab is considered less toxic and better tolerated than cyclophosphamide. Therefore, rituximab is the preferred choice for remission induction [16]. Achievement of complete remission with this regimen of rituximab and corticosteroids may take several months. Therefore, patients should be monitored on this regimen before they are considered to be non-responders. Remission is indicated by the resolution of inflammatory manifestations [2]. Cyclophosphamide is usually reserved for the treatment of active disease that does not respond to rituximab [16].

Nonsevere GPA is characterized by vasculitis without life- or organ-threatening manifestations. In the treatment of non-severe GPA, methotrexate is preferred over cyclophosphamide or rituximab for remission induction as it is considered less toxic [16].

In patients with active remission after newly diagnosed GPA, the choice of therapy is influenced by disease severity and patient factors. Rituximab is the suggested treatment for remission maintenance. Studies show a lower relapse rate with rituximab. However, methotrexate and azathioprine are equally efficacious in remission maintenance. Mycophenolate mofetil (MMF) is also considered an alternative therapy. The duration of remission maintenance therapy is usually 18 months or longer and depends on the clinical condition and patient preference [16].

## Conclusions

Early identification and prompt treatment are mandatory in managing patients with Wegener's granulomatosis. In this index case, since the patient presented in the early course of the disease, a satisfactory improvement in her clinical status could be achieved.
